# Prevalence and Causes of Myocardial Infarction with Non-Obstructive Coronary Arteries in a Contemporary Cohort of Patients with Suspected Myocardial Infarction

**DOI:** 10.3390/jcm10215188

**Published:** 2021-11-06

**Authors:** Dominik Dees, Faridun Rahimi, Michael Amann, Thomas G. Nührenberg, Nikolaus Löffelhardt, Roland Schmitz, Christian M. Valina, Franz-Josef Neumann, Willibald Hochholzer

**Affiliations:** Department of Cardiology and Angiology II, University Heart Center Freiburg Bad Krozingen, 79189 Bad Krozingen, Germany; faridun.rahimi@uniklinik-freiburg.de (F.R.); Michael.amann@uniklinik-freiburg.de (M.A.); thomas.nuehrenberg@uniklinik-freiburg.de (T.G.N.); nikolaus.loeffelhardt@uniklinik-freiburg.de (N.L.); roland.schmitz@uniklinik-freiburg.de (R.S.); christian.valina@uniklinik-freiburg.de (C.M.V.); franzjosef.neumann@uniklinik-freiburg.de (F.-J.N.); willibald.hochholzer@universitaets-herzzentrum.de (W.H.)

**Keywords:** MINOCA, myocardial infarction, non-obstructive coronary arteries, predictors

## Abstract

Background: A significant proportion of patients presenting with acute myocardial infarction (MI) has no coronary obstruction at coronary angiography and no other obvious non-coronary pathophysiology causing MI. These patients are classified as MI with non-obstructive coronary arteries (MINOCA). Data on incidence and predictors of MINOCA are still limited. Methods: This study enrolled patients presenting symptoms suggestive of MI and undergoing a comprehensive cardiac work-up including an early invasive strategy. Patients with non-obstructive coronary arteries and without other obvious reasons for MI were scheduled for further work-up including magnetic resonance or intraluminal imaging. MINOCA was diagnosed according to the current European Society of Cardiology guidelines. Results: From the 1532 patients enrolled, 730 had available coronary imaging and 546 were diagnosed with MI. No significant coronary obstructions were found in 117 patients with MI. After the exclusion of 6 patients with acute myocarditis or takotsubo-syndrome as well as 88 with type II MI, 23 patients were diagnosed with MINOCA (4% of all MIs). Among these 23 patients, the most common etiology of MINOCA was thromboembolic events followed by coronary spasm. Female sex, the absence of hypercholesterolemia, and a normal left-ventricular ejection fraction were independently predictive for MINOCA compared to patients with other causes of MI. Conclusion: More than 20% of patients presenting with acute MI showed no significant coronary obstruction. About 4% of these patients were diagnosed with MINOCA. Female sex, a lower cardiovascular risk profile, and normal left-ventricular function were predictive for MINOCA.

## 1. Introduction

Approximately 5–10% of all patients presenting with acute myocardial infarction (MI) have no significant coronary obstruction [1,2,3]. The current 4th Universal Definition of Myocardial Infarction labeled this entity as myocardial infarction with non-obstructive coronary arteries (MINOCA) and defined it as MI without ≥50% diameter stenosis in any major epicardial vessel [4].

The diagnosis of MINOCA also requires the myocardial injury to be caused by an ischemic mechanism and that non-ischemic causes such as myocarditis be excluded [4]. The etiology of MINOCA is diverse. Potential pathophysiologic mechanisms include intracoronary plaque disruption or erosion, coronary spasm, spontaneous coronary dissection, or coronary embolism. Several studies demonstrated that MINOCA is not a benign condition [5,6] given an annual mortality of up to 5% [7].

The diagnosis of MINOCA is challenging and multiple definitions have been published in recent years [4,8,9]. The current 2020 European Society of Cardiology (ESC) guidelines for the management of acute coronary syndromes in patients presenting without persistent ST-segment elevation (NSTE-ACS) provide a clear and strict definition of MINOCA [10]. To establish the diagnosis of MINOCA, a comprehensive work-up is recommended to exclude alternate diagnoses for the clinical presentation. Non-coronary etiologies such as myocarditis, takotsubo-syndrome, and type II MI need to be ruled out and, compared to previous definitions, the diagnosis of MINOCA is limited to coronary causes such as coronary spasm or coronary dissection.

Real-world data on MINOCA from large cohorts as well as data on potential risk factors for this syndrome are still limited but might help identify patients at risk.

Thus, this analysis sought to investigate the prevalence and predictors of MINOCA in a large cohort of patients presenting with suspected acute MI.

## 2. Methods

### 2.1. Study Design

This study is a secondary analysis of the FAST-MI study, which was a prospective cohort study enrolling patients with suspected acute MI at the chest pain unit of a tertiary care heart center [11]. The aim of this study was the identification and validation of algorithms for a fast diagnosis of MI. Patients were enrolled from November 2015 until December 2016.

Key inclusion criteria were being ≥18 years in age and presenting with either typical clinical symptoms suggestive of acute MI starting within the last 24 h before enrollment, or new typical ECG changes or typical imaging findings. Key exclusion criteria were missing troponin test results, subacute symptoms lasting for more than 24 h, or a previously diagnosed or ruled-out acute MI in a referring hospital. The study was approved by the ethics committee of the University of Freiburg (Germany; German Clinical Trials Register, https://www.drks.de/drks_web/ (accessed on 4 November 2021), Identifier: DRKS00009713).

The present analysis used the extended data set of FAST-MI [11], which contains patients that were excluded from the primary analysis due to criteria identified only after enrollment (e.g., symptoms lasting for more than 24 h or established diagnosis of MI before admission). Patients not undergoing coronary imaging for multiple reasons were excluded from this analysis since this criterion is needed for the diagnosis of MINOCA.

### 2.2. MINOCA Work-Up

All patients underwent a comprehensive clinical assessment according to the treatment algorithms of our heart center, including physical examination, routine blood tests, 12-lead ECG, continuous monitoring, and early echocardiography (within 3 h). Levels of high-sensitivity cardiac troponin T (hs-cTnT) were measured at presentation, after 1 h, and thereafter if clinically indicated. All blood samples were immediately assayed in our central laboratory.

**Figure 1:**
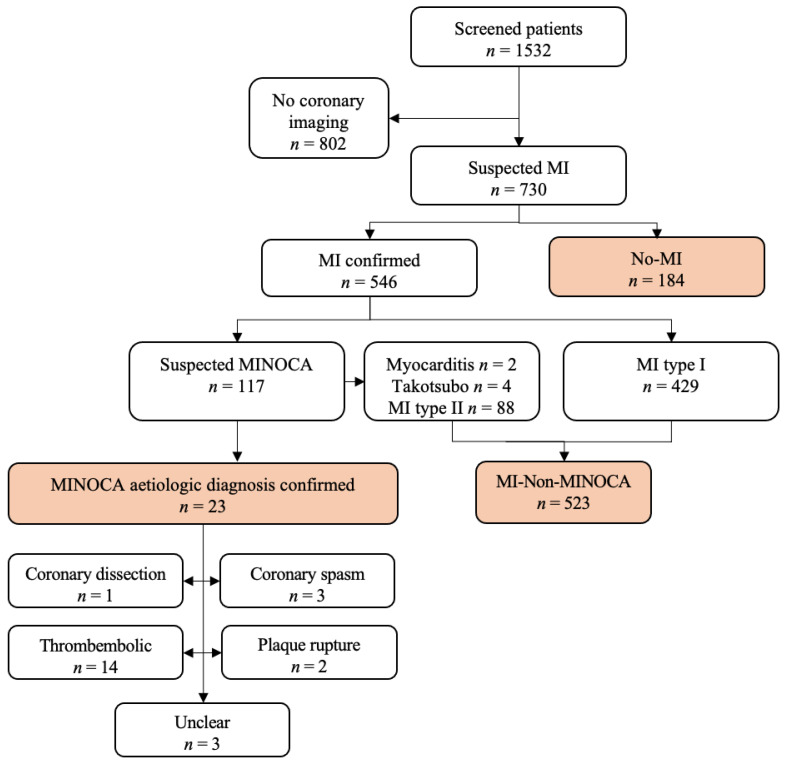
Study flow. MI, myocardial infarction; MINOCA, MI with non-obstructive coronary arteries.

Patients were classified according to the current ESC NSTE-ACS guideline from 2020 [10] and the 4th Universal Definition of Myocardial Infarction [4] (Figure 1). For the diagnosis of MI, patients had to fulfill the criteria of the 4th Universal Definition, needing an acute myocardial injury (elevated cardiac troponin values with at least one value above the 99th percentile upper reference limit and a rise and/or fall of values) with clinical evidence of acute myocardial ischemia (typical symptoms and/or typical ECG changes and/or typical imaging findings).

**Figure 2:**
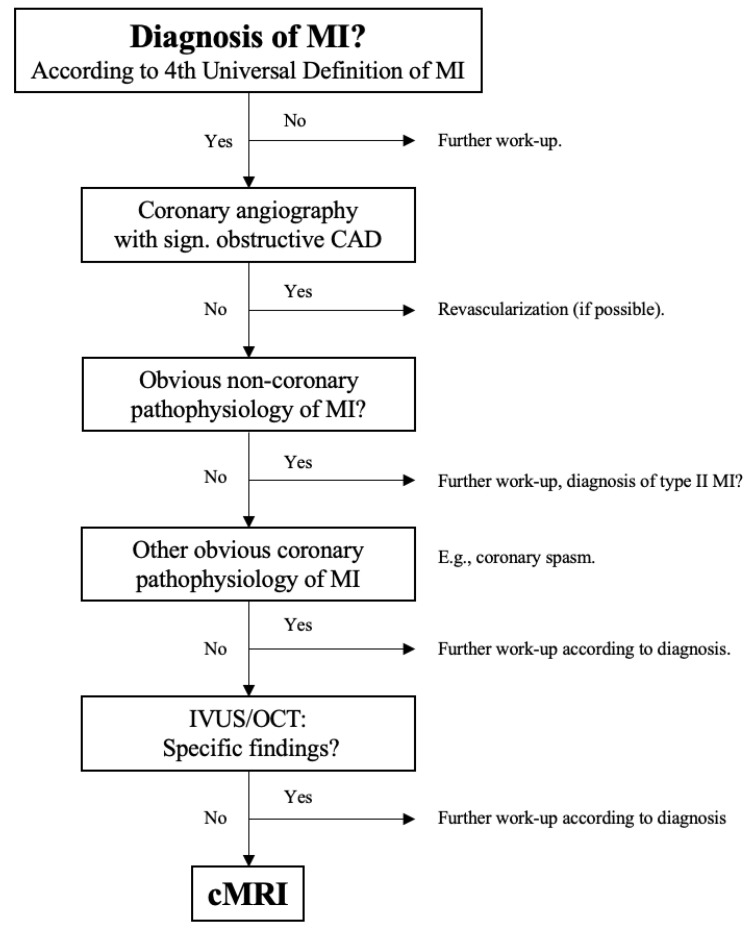
Diagnostic work-up of patients with MI. CAD, coronary artery disease; MI, myocardial infarction; IVUS, intravascular ultrasound; OCT, optical coherence tomography; cMRI, cardiac magnetic resonance imaging.

Figure 2 shows the diagnostic algorithm for patients diagnosed with acute MI. All patients included in the present analysis underwent coronary imaging mainly by invasive coronary angiography. If an obstructive coronary artery disease caused by atherosclerosis (i.e., stenosis ≥ 50% in any potential infarct-related artery) was found, patients underwent revascularization if possible (by percutaneous coronary intervention in many patients). All other patients underwent further investigation if there was no obvious non-coronary cause for MI (e.g., sepsis) or another coronary pathophysiology such as coronary spasm apparent.

As recommended by the ESC NSTE-ACS guideline from 2020, intracoronary imaging (mainly OCT) was used in the remaining patients to rule out another coronary pathophysiology such as coronary dissection. All patients remaining with an uncertain cause of MI as well as patients with symptoms suggestive for myocarditis, takotsubo-syndrome, or another cardiomyopathy underwent cardiac magnetic imaging (MRI) if clinically reasonable and tolerable for the patient. Further investigations were performed according to clinical presentation (e.g., transesophageal echocardiography in case of suspected coronary embolism).

The final diagnosis of MINOCA was based on the 2020 ESC NSTE-ACS guideline excluding patients with type II MI, myocarditis, takotsubo-syndrome, or other cardiomyopathies.

### 2.3. Statistical Analysis

Patients were stratified into MINOCA, all other types of MI (MI-non-MINOCA), and patients without MI. Categorical variables are presented as percentages and compared by a chi-square-test. Continuous variables are presented by median with interquartile range and compared by the Kruskal–Wallis test. The association of variables with MINOCA (vs. MI-Non-MINOCA) was tested with binary logistic regression analyses. All variables with a *p*-value of <0.05 in univariable regression analyses were used for the multivariable model. An alpha-level of <0.05 was regarded as statistically significant.

## 3. Results

### 3.1. Patient Classification and Characteristics

This study enrolled 1532 patients presenting with symptoms suggestive of acute MI and a planned early invasive strategy. For the present analysis, 802 patients without coronary imaging were excluded (Figure 1). In most cases, the reason for omitting the early invasive strategies was an obvious non-coronary disease following initial evaluation (e.g., pneumonia without any pathological cardiac findings at non-invasive testing) or patient refusal. From the 730 patients with coronary imaging, 546 were diagnosed with MI and 184 patients with unstable angina or diagnoses other than MI according to the 4th Universal Definition of MI. From 546 patients fulfilling the diagnostic criteria of MI, 429 were classified as type I MI due to coronary obstruction caused by atherothrombotic coronary artery disease and precipitated by atherosclerotic plaque disruption (rupture or erosion). A total of 88 patients were diagnosed as type II MI caused by a disbalance between oxygen supply and demand. The pathophysiologies causing these type II MIs were hypertensive emergencies in 20 patients, acute heart failure in 28 patients (mainly associated with valvular heart disease), atrial tachycardia in 22 patients, sepsis in 12 patients, acute renal failure in 4 patients, and pulmonary embolism in 2 patients.

In six patients, further work-up revealed a non-coronary reason for myocardial necrosis: two patients were diagnosed with acute myocarditis based on typical signs and MRI findings and four with takotsubo-syndrome. These patients were not classified as MINOCA according to the 2020 ESC NSTE-ACS guideline and were assigned the MI-Non-MINOCA group (Figure 1).

The remaining 23 patients fulfilled the diagnostic criteria for MINOCA according to the 2020 ESC NSTE-ACS guideline, which represents a proportion of 4.2% of all MI in the investigated cohort.

Further diagnostic work-up including intravascular imaging and cardiac MRI revealed the pathomechanism in most of the remaining 23 patients with MINOCA: 2 patients presented with a non-obstructive plaque rupture, 1 patient with a spontaneous coronary dissection, 3 patients with coronary spasm, and 14 patients with a thromboembolic coronary occlusion despite otherwise normal coronary arteries.

In three patients with proven acute, focal myocardial necrosis by MRI, the pathophysiology of MI could not be clarified (Figure 1). All the patients except the patients with coronary hyperresponsiveness received further intraluminal imaging (mainly by OCT).

Compared to the other patients, patients with MINOCA were less likely to be male and had a lower prevalence of diabetes mellitus, arterial hypertension, and hypercholesterolemia (Table 1).

Furthermore, patients with MINOCA had significantly less often a history of previous PCI or peripheral artery disease and were less frequently treated with aspirin. Chest pain was the main leading symptom in patients with MINOCA.

The results of the diagnostic workup are shown in Table 2. Patients with MINOCA showed significantly lower levels of cardiac troponin and urea, less often ST-segment changes, and had an impaired LV-function less often compared to patients with MI with other causes.

### 3.2. Predictors for MINOCA

Binary logistic regression analyses were used to identify predictors of MINOCA in the group of patients diagnosed with MI. Several variables were significantly associated with MINOCA in univariable analyses (Table 3). From these variables, only few prevailed as independent predictors for MINOCA in the multivariable model. Female sex, the absence of hypercholesterolemia, and a normal left-ventricular function were independently predictive of MINOCA compared to patients with MI with other causes.

## 4. Discussion

This cohort study sought to investigate the prevalence and predictors of MINOCA in an unselected cohort of patients presenting with suspected acute MI and planned early invasive strategy. The diagnosis of MINOCA has been a focus of cardiology in recent years given the finding that some patients presenting with signs very suggestive of an acute coronary obstruction show only minimal or no coronary heart disease at time of angiography. Several statements of cardiac societies addressed this topic, including position papers and the 2020 current NSTE-ACS guideline [8,10].

The key findings of this study can be summarized as follows: more than 20% of patients presenting with acute MI showed no significant coronary obstruction at the time of angiography. However, when excluding patients with type II MI and other non-coronary pathologies, this number drops to about 4%. Independent variables predictive of MINOCA in patients presenting with MI were female sex, the absence of hypercholesterolemia, and normal left ventricular function.

In the present study, MINOCA was defined according to the current ESC NSTE-ACS guidelines [10]. This definition only includes patients with non-coronary causes and excludes non-coronary pathophysiologies such as myocarditis or type II MI, which is in line with a previous scientific statement from the American Heart Association [9]. Previous definitions, for example the ESC position paper on MINOCA [8], also included patients with non-coronary causes of MI, usually with myocardial cell necrosis caused by a supply–demand mismatch.

However, there is still no international consensus on how to define MINOCA, and multiple definitions have already been proposed. In 2017, a position paper from the ESC defined MINOCA more liberally including patients with type II MI as well as patients with takotsubo-syndrome but excluding patients with myocarditis and cardioembolism [8]. Another statement from 2017 used a different definition [12]. Patients with troponin elevation and non-obstructive coronary arteries were labeled as patients with “troponin positive non-obstructive coronary arteries” with three major subgroups: myocardial disorders, such as cardiomyopathies, takotsubo, or myocarditis; non-cardiac disorders, such as pulmonary embolism or renal impairment; and coronary disorders, which represented MINOCA.

The lacking common definition for MINOCA might explain the different incidences seen in various cohorts. In previous studies, the prevalence of MINOCA ranged between 5% and10% [1,13]. A recent study from Dreyer et al. identified 6% of patients in their cohort with MINOCA [14]. The inclusion of type II MI in previous definitions for MINOCA [8] might have led to higher prevalences of MINOCA in previous studies [3,13,15]. In the present analysis, the combined prevalence of patients with type II MI and MINOCA would have been about 20%. The relatively low prevalence of MINOCA in the present cohort is most likely the result of a very stringent work-up of patients with MI but non-obstructive coronary arteries. The wide use of MRI most likely helped to identify patients with other cardiac diseases such as myocarditis that might have otherwise been incorrectly labeled as MINOCA.

The present analysis identified several variables independently associated with MINOCA. The negative predictive value of cardiovascular risk factors for MINOCA (but positive predictive value for MI-Non-MINOCA) appears plausible. Independent variables found to be predictive for MINOCA were female sex and absence of hypercholesterolemia. The association of MINOCA with female sex as well as with lower levels of LDL cholesterol is in line with previous studies [7].

The analysis revealed a significant difference in left-ventricular ejection fraction in patients with MINOCA compared to patients with MI-Non-MINOCA. Patients with MINOCA showed a significantly better ejection fraction than patients with MI-Non-MINOCA. This result was also reported in recent studies [13,16]. However, this finding does not imply that MINOCA is a benign condition [6], given an annual mortality rate of up to 5% [7]. Another interesting finding was that most patients with MINOCA were diagnosed with a thromboembolic coronary event. Taken together with the lower prevalence of cardiovascular risk factors in patients with MINOCA, this points to mechanisms other than atherosclerosis causing this condition. Potential mechanisms could be cardiac fibrosis, which is an established risk factor for thromboembolic events or other structural changes such as patent foramen ovale.

## 5. Limitations

The present study is observational in nature with all adherent limitations. Furthermore, due to clinical reasons, not all patients underwent MRI testing. Pharmacological reactivity testing for provocation of coronary spasm was not performed. Thus, it cannot be excluded that additional testing would have changed the classification of patients. A clinical follow-up was only available in a limited proportion of patients and therefore not reported.

## 6. Conclusions

More than 20% of patients presenting with acute MI showed no significant coronary obstruction. About 4% of these patients were diagnosed with MINOCA. Female sex and a lower cardiovascular risk profile as well as a normal left-ventricular function were predictive for MINOCA.

## Figures and Tables

**Table 1:** Baseline characteristics.

	MINOCA *n* = 23	MI-Non-MINOCA *n* = 523	No-MI *n* = 184	*p*-Value
Age, years	63.2 {54.0–7.0}	69.8 {62.0–79.0}	68.9 {61.0–78.0}	0.81
Male	12 (52.2%)	389 (74.4%)	114 (62.0%)	0.001
Body mass index, kg/m^2^	26.5 {24.6–28.3}	27.7 {24.7–29.7}	27.9 {24.7–30.3}	0.36
**Cardiovascular risk factors**				
Arterial hypertension	12 (52.2%)	437 (83.6%)	155 (84.2%)	<0.001
Diabetes mellitus	1 (4.3%)	161 (30.8%)	37 (20.1%)	0.001
Hypercholesterolemia	10 (43.5%)	394 (75.3%)	133 (72.3%)	0.003
Current smoking	3 (13.0%)	120 (22.9%)	32 (17.4%)	0.12
**Medical history**				
Previous PCI	4 (17.4%)	232 (44.4%)	75 (40.7%)	0.03
Previous CABG	0 (0%)	57 (10.9%)	12 (6.5%)	0.06
Previous myocardial infarction	4 (17.4%)	125 (12.9%)	38 (20.6%)	0.54
Congestive heart failure	0 (0%)	25 (4.8%)	7 (3.8%)	0.49
Peripheral artery disease	0 0%)	64 (12.2%)	13 (7.1%)	0.04
Previous Stroke/TIA	1 (4.3%)	53 (2.8%)	12 (6.5%)	0.25
Previous severe bleeding	0 (0%)	15 (2.6%)	9 (4.8%)	0.28
Chronic pulmonary disease	0 (0%)	38 (7.2%)	21 (11.4%)	0.07
**Leading symptom**				0.46
Chest pain	19 (82.6%)	376 (71.9%)	126 (68.5%)	
Dyspnea	1 (4.3%)	88 (16.8%)	32 (17.4%)	
Collapse	0 (0%)	13 (2.5%)	8 (4.3%)	
Others	3 (13.0%)	46 (8.7%)	14 (7.6%)	
**Medication on admission**				
Aspirin	6 (26.1%)	282 (53.9%)	82 (44.6%)	0.005
P2Y12-receptor inhibitor	0 (0%)	62 (11.9%)	29 (15.8%)	0.07
Oral anticoagulants	2 (8.7%)	107 (20.5%)	48 (26.1%)	0.09
ß-Blocker	7 (30.4%)	254 (48.6%)	98 (53.2%)	0.11
Nitrates	0 (0%)	32 (6.1%)	8 (4.3%)	0.33
Statin	7 (30.4%)	257 (49.3%)	90 (48.9%)	0.83

Continuous variables are presented as mean (interquartile range) and compared by the Kruskal–Wallis test. Categorial variables are presented as a percentage and compared by a chi-square test. MI, myocardial infarction; PCI, percutaneous coronary intervention; CABG, coronary artery bypass grafting; TIA, transient ischemic attack.

**Table 2:** Diagnostic findings.

	MINOCA *n* = 23	MI-Non-MINOCA *n* = 523	No MI *n* = 184	*p*-Value
**Baseline blood testing**				
High-sensitive troponin T, ng/mL	0.11 {0.02–0.45}	0.26 {0.02–0.51}	0.01 {0.007–0.03}	<0.001
LDL-cholesterol, mg/dL	117 {39–183}	119 {30–359}	108 {41–230}	0.22
Cholesterol, mg/dL	182 {106–280}	183 {150–450}	175 {143–297}	0.99
Creatinine, mg/dL	1.0 {0.6-1.5}	1.2 {0.4-6.6}	1.1 {0.4-6.2}	0.98
Urea, mg/dL	39 {21–77}	43 {16–187}	41.0 {30–143}	0.02
Glomerular filtration rate, mL/min	76 {41–115}	75 {9–178}	76 {61–172}	0.87
Hemoglobin, g/dL	14 {11–17}	13 {6–19}	14 {13–18}	0.99
C-reactive protein, mg/dL	1.3 {0.3–11.2}	1.5 {0.3–24.0}	0.8 {0.3–16.6}	0.46
**ECG at baseline**				
Initial rhythm				0.62
Sinus rhythm	21 (91.3%)	452 (86.4%)	154 (83.7%)	
Atrial fibrillation	2 (8.7%)	57 (10.9%)	25 (13.6%)	
Ventricular tachycardia	0 (0%)	0 (0.0%)	1 (0.5%)	
Supraventricular tachycardia	0 (0%)	4 (0.8%)	0 (0.0%)	
ST-segment changes	4 (17.4%)	102 (19.5%)	12 (6.5%)	<0.001
T-wave inversion	3 (13.0%)	87 (16.6%)	20 (10.9%)	0.16
Bundle branch block	4 (17.4%)	92 (17.6%)	28 (15.2%)	0.76
**Echocardiography**				
Impaired left ventricular function	2 (8.7%)	259 (49.5%)	52 (9.9%)	<0.001
Moderate/severe aortic stenosis	1 (0.5%)	32 (6.1%)	12 (6.5%)	0.92
Moderate/severe aortic insufficiency	1 (3.6%)	8 (1.2%)	5 (2.7%)	0.41
Moderate/severe mitral insufficiency	1 (4.3%)	57 (10.9%)	20 (10.9%)	0.61

Continuous variables are presented as mean (interquartile range) and compared by the Kruskal–Wallis test. Categorial variables are presented as a percentage and compared by a chi-square test. MI, myocardial infarction; ECG, electrocardiogram.

**Table 3:** Binary logistic regression analyses for MINOCA versus MI-Non-MINOCA.

	Univariable Analyses			Multivariable Analysis		
Diagnosis	OR	95% CI	*p*-Value	OR	95% CI	*p*-Value
Age per year	0.96	(0.93–0.99)	0.01	0.98	(0.94–1.02)	0.18
Female Sex	2.67	(1.15–6.18)	0.02	3.33	(1.24–8.93)	0.02
**Cardiovascular risk factors**						
Arterial hypertension	0.21	(0.09–0.50)	<0.001	0.56	(0.20–1.57)	0.27
Diabetes mellitus	0.10	(0.01–0.76)	0.03	0.16	(0.02–1.28)	0.08
Hypercholesterolemia	0.25	(0.11–0.59)	0.002	0.36	(0.14–0.95	0.04
Current smoking	0.51	(0.15–1.73)	0.28			
**Medical history**						
Previous PCI	0.27	(0.09–0.79)	0.02	0.98	(0.22–4.29)	0.98
Previous CABG		not in group				
Previous MI	0.67	(0.22–2.01)	0.48			
Congestive heart failure		not in group				
Previous stroke/TIA	0.40	(0.05–3.06)	0.38			
Previous severe bleeding		not in group				
Chronic pulmonary disease		not in group				
**Leading symptom**						
Chest pain	0.22	(0.03–1.68)	0.15			
Dyspnea		not in group				
Collapse	1.29	(0.37–4.53)	0.69			
**Medicaments on admission**		not in group				
Aspirin	0.30	(1.12–0.78)	0.01	0.80	(0.23–2.86)	0.73
Oral anticoagulant	0.37	(0.09–1.59)	0.18			
ß-Blocker	0.46	(0.19–1.14)	0.09			
Nitrates		not in group				
Statins	0.45	(0.18–1.12)	0.09			
**Baseline blood test results**						
High-sensitivity Troponin T per 1 ng/mL	0.98	(0.57–1.70)	0.95			
Hemoglobin per 1 g/L	1.10	(0.88–1.38)	0.40			
Glomerular filtration rate per 1 mL/min	0.99	(0.98–1.01)	0.83			
C-reactive protein per 1 mg/L	0.98	(0.84–1.13)	0.77			
LDL-cholesterol per 1 mmol/L	1.00	(0.99–1.01)	0.81			
**ECG**						
Atrial fibrillation	0.59	(0.14–2.60)	0.49			
ST-segment changes	0.87	(0.29–2.62)	0.81			
T-wave inversion	0.75	(0.22–2.59)	0.65			
Bundle branch block	1.01	(0.33–2.97)	0.98			
**Echocardiography**						
Impaired left ventricular function	0.09	(0.02–0.42)	0.002	0.14	(0.03–0.61)	0.009
Moderate/Severe aortic stenosis	0.69	(0.09–5.35)	0.73			
Moderate/Severe mitral insufficiency	0.37	(0.05–2.82)	0.33			

PCI, percutaneous coronary intervention; CABG, coronary artery bypass grafting; ECG, electrocardiogram; MI, myocardial infarction; TIA, transient ischemic attack. All variables with a *p*-value < 0.05 in univariable regression analyses were selected for the multivariable model.

## Data Availability

The study does not provide publicly available datasets.
